# Prevalence and Risk Factors of Acute Pancreatitis in Childhood Acute Leukemia

**DOI:** 10.3390/cancers18060910

**Published:** 2026-03-11

**Authors:** Kamonluk Thepuatrakul, Atchariya Chanpong, Natsaruth Songthawee, Pornpun Sripornsawan, Sirinthip Kittivisuit, Hansa Sriphongphankul, Thirachit Chotsampancharoen

**Affiliations:** 1 Department of Pediatrics, Faculty of Medicine, Prince of Songkla University, Songkhla 90110, Thailand; 2 Division of Gastroenterology and Hepatology, Department of Pediatrics, Faculty of Medicine, Prince of Songkla University, Songkhla 90110, Thailand; 3 Division of Hematology and Oncology, Department of Pediatrics, Faculty of Medicine, Prince of Songkla University, Songkhla 90110, Thailand

**Keywords:** pancreatitis, leukemia, children

## Abstract

Background/Objectives: Acute pancreatitis (AP) is an uncommon but serious complication in children undergoing treatment for acute leukemia. We aimed to determine the prevalence of AP in pediatric patients with acute leukemia, identify its risk factors, and evaluate their impact on treatment outcomes and overall survival. Materials and Methods: We retrospectively reviewed the medical records of children with acute leukemia who developed acute abdominal pain suggestive of AP at Songklanagarind Hospital between 2004 and 2024. Demographic data, including leukemia subtypes, treatment protocols, and clinical outcomes, were compared between the patients with and without AP. Results: Of the 618 patients with leukemia, 70 children with abdominal pain were identified, and 17 were diagnosed with AP. The prevalence of AP was 2.8%. Most children with acute leukemia and AP had T-cell subtypes (50.0%) and received high- to very-high-risk treatment protocols (76.5%). Patients with AP experienced a shorter duration of abdominal pain before diagnosis and required imaging more frequently than the non-AP patients did (100% vs. 56.6%). They required a prolonged fasting period and greater intravenous fluid volume within 48 h. The overall mortality rate (all-cause during follow-up) was significantly higher in the AP group. Using high- to very-high-risk chemotherapy protocols was a risk factor for AP, and the accumulative L-asparaginase dose of ≥55,200 IU/m^2^ could increase AP risk. Conclusions: AP is significantly associated with increased overall mortality in children with acute leukemia. Careful monitoring of L-asparaginase dosing may be required. Larger studies are needed to better identify the risk factors and preventive strategies.

## Simple Summary

Acute pancreatitis (AP) is a rare but serious complication in children receiving treatment for acute leukemia. We retrospectively reviewed cases from 2004 to 2024 at Songklanagarind Hospital to determine prevalence, risk factors, and outcomes. Among 618 patients, 17 (2.8%) were diagnosed with AP, which was more common in patients with T-cell leukemia and in those receiving high- to very-high-risk chemotherapy. Compared with patients without AP, those with AP required more imaging studies, prolonged fasting, and greater intravenous fluid support. Overall mortality was significantly higher in the AP group. An increased AP risk was associated with the use of high- to very-high-risk treatment protocols and a cumulative L-asparaginase dose ≥55,200 IU/m^2^. Three patients received L-asparaginase after AP resolved; all were high- to very-high-risk cases, and one developed persistent AP with walled-off necrosis. This finding may indicate genetic susceptibility, although genetic testing was not performed. Careful monitoring of L-asparaginase dosing is recommended.

## 1. Introduction

Acute leukemia is the most common childhood malignancy, with survival rates of 80–90% in many settings [1]. Treatment-related complications are among the major contributors to morbidity and mortality worldwide. Acute pancreatitis (AP) remains an important complication in pediatric oncology [2,3]. AP diagnosis is established based on the presence of characteristic abdominal pain, increased serum pancreatic enzyme levels, and supportive findings on imaging studies [4].

The pathophysiology of AP involves the premature activation of pancreatic digestive enzymes (particularly trypsin) within acinar cells, leading to autodigestion, local inflammation, vascular injury, and, in severe cases, systemic inflammatory responses [3]. Chemotherapy used in pediatric oncology may cause direct acinar toxicity, hypersensitivity reactions, mitochondrial injury, or metabolic disturbances, such as dyslipidemia, which could increase the risk of pancreatitis [5]. Patients may present with a range of clinical manifestations, from minor abdominal pain to multi-organ involvement [6].

The reported incidence of AP during leukemia therapy varies from 2.3% to 18% in international cohorts [2,7,8,9,10,11]. Direct mortality is relatively low; however, AP has the potential to seriously impede leukemia treatment by necessitating the postponement or permanent discontinuation of vital chemotherapeutic agents, which can increase the chance of relapse [2,9,12]. Children affected by AP can develop acute or chronic complications such as insulin dependence and pancreatic pseudocysts [2]. Older age and intensity of treatment have been reported as predictors of worsening outcomes [2,9,10,12].

However, most current evidence is derived from Western studies. In Thailand, available and up-to-date data are relatively limited, with few reports originating from the central region [10]. Notably, there is a paucity of data on AP in pediatric leukemia cases in Thailand, particularly in Southern Thailand, where toxicity profiles may be influenced by access to healthcare, demographics, and updated chemotherapy protocols. Substantial regional gaps in knowledge remain; accordingly, in this study, we sought to examine the epidemiology and clinical course of AP in children undergoing treatment for acute leukemia.

The aim of this study was to determine the incidence of AP in children treated for acute leukemia, identify associated risk factors, and evaluate their impact on treatment outcomes and overall survival.

## 2. Materials and Methods

Medical records of children ≤15 years old, diagnosed with acute leukemia at Songklanagarind Hospital between 2004 and 2024, were retrospectively reviewed. Pediatric patients diagnosed with acute leukemia who presented with acute abdominal pain thought to be caused by AP were included. To ensure the diagnosis of AP, the patient’s clinical history, laboratory tests, and imaging examinations were reviewed. This study was approved by the Ethics Committee of the Faculty of Medicine, Prince of Songkla University (REC. 59-107-01-1). The requirement for informed consent was waived owing to the retrospective nature of the study and use of de-identified data.

### 2.1. Data Collection and Definitions

Demographic data, clinical manifestations, diagnosis, clinical course of acute leukemia, previous investigations, medications administered, particularly chemotherapy, and outcomes were collected. The cumulative dose of L-asparaginase was defined as the total administered dose per body surface area (BSA), calculated from treatment initiation until the onset of abdominal pain. The cumulative dose was manually calculated based on the most recent BSA, using the measurement closest to the onset of abdominal pain, and the same method was applied to all patients. Fluids in the first 48 h referred to the total intravenous fluid requirement within 48 h after presentation with abdominal pain. Diagnostic amylase and lipase levels refer to the serum levels measured at the time of presentation with abdominal pain, whereas peak levels represent the maximum amylase and lipase concentrations observed during pain episodes.

AP was diagnosed according to the Atlanta criteria, as modified by the INSPPIRE group for pediatric patients [13,14]. The diagnosis required the presence of at least two of the following three criteria:

- Abdominal symptoms consistent with acute pancreatitis;
- Serum amylase or lipase levels elevated to at least three times the upper limit of normal;
- Imaging evidence of acute pancreatitis on ultrasonography (USG), computed tomography (CT), or magnetic resonance imaging (MRI).

Wondmeneh TG et al. reported that the severity of acute pancreatitis was graded according to version 6.0 of the Common Terminology Criteria for Adverse Events (CTCAE v.6.0) as follows [15,16]:

- Grade 1: No specific definition provided.
- Grade 2: Elevated levels of pancreatic enzymes or abnormalities detected only on imaging.
- Grade 3: Severe abdominal pain and vomiting; medical intervention indicated (e.g., analgesia, nutritional support).
- Grade 4: Life-threatening consequences; urgent intervention required.
- Grade 5: Death.

### 2.2. Chemotherapy Protocols

For patients with acute lymphoblastic leukemia (ALL) treated between 2004 and 2005, chemotherapy protocols were based on the Children’s Cancer Study Group (CCG) criteria, including CCG-104, CCG-105, and CCG-106 for patients with good, intermediate, and poor prognosis, respectively [17,18,19,20]. Between 2006 and 2013, the Thai national protocol, including ALL-01-05, ALL-02-05, and ALL VHR-08 were implemented for patients with standard-, high-, and very high-risk ALL, respectively [19,21,22]. Notably, the latest version of the Thai national protocol, modified from the Children’s Oncology Group (COG)-AALL00P2 and COG-AALL0232 regimens, was used between 2014 and 2024. These included ALL 1301, ALL 1302, and ALL 1303 for patients with standard-, high-, and very high-risk ALL, respectively [19,23]. Notably, the risk stratification at presentation was determined according to the National Cancer Institute risk group classification [19,24,25].

For patients with acute myeloid leukemia (AML), those treated before 2007 received the AML–Berlin–Frankfurt–Munster (BFM)-83 protocol, whereas patients treated between 2008 and 2013 received the AML-BFM-98 protocol [26,27,28]. Between 2014 and 2024, patients with AML were treated using the Thai national protocol adapted from the COG, using AML-1301 and AML-1302 for standard-risk and high-risk AML, respectively [27,29,30].

All patients received native L-asparaginase because it was the only formulation available at our institution.

### 2.3. Statistical Analysis

All analyses were conducted using R (version 4.4.3; R Foundation for Statistical Computing, Vienna, Austria). Baseline characteristics are reported as median (interquartile range [IQR]), mean ± standard deviation (SD), or number (percentage), as appropriate. Continuous and categorical variables were compared using Fisher’s exact test, the Mann–Whitney U test, or the Kruskal–Wallis test, as applicable.

Univariable and multivariable logistic regression analyses were performed to examine associations between potential risk factors and AP diagnosis. Variables with a *p* value < 0.2 in the univariable analysis were included in the multivariable model. Logistic regression results are presented as odds ratios (ORs) with 95% confidence intervals (CIs). Variance inflation factors were calculated to assess multicollinearity among the variables in the multivariable models.

Receiver operating characteristic (ROC) curve analysis was used to evaluate the diagnostic accuracy of continuous variables. Kaplan–Meier survival analysis was applied to estimate the time to AP occurrence across different leukemia subtypes. *p* < 0.05 was considered statistically significant.

## 3. Results

### 3.1. Patient Characteristics

Of the 853 children referred to Songklanagarind Hospital and diagnosed with acute leukemia, 618 were treated with chemotherapy and followed up at our center; 50.6% had B-cell ALL, 10.0% had T-cell ALL, 12.9% had French–American–British-classified acute lymphoblastic leukemia (FAB-ALL), and 26.4% had AML. Among the 618 children, 70 were evaluated for suspected AP (presenting with abdominal pain), of whom 17 were diagnosed with AP, representing 24.3% of patients who presented with abdominal pain. Of the 17 patients with AP, 6 had T-cell ALL, 8 had B-cell ALL, 3 had AML, and none had FAB-ALL. The overall AP prevalence was 2.8%. Among the 17 AP cases, the majority (65%) were classified as moderate to severe (CTCAE grades 3–5) pancreatitis, and three patients died because of the AP episode.

**Figure 1:**
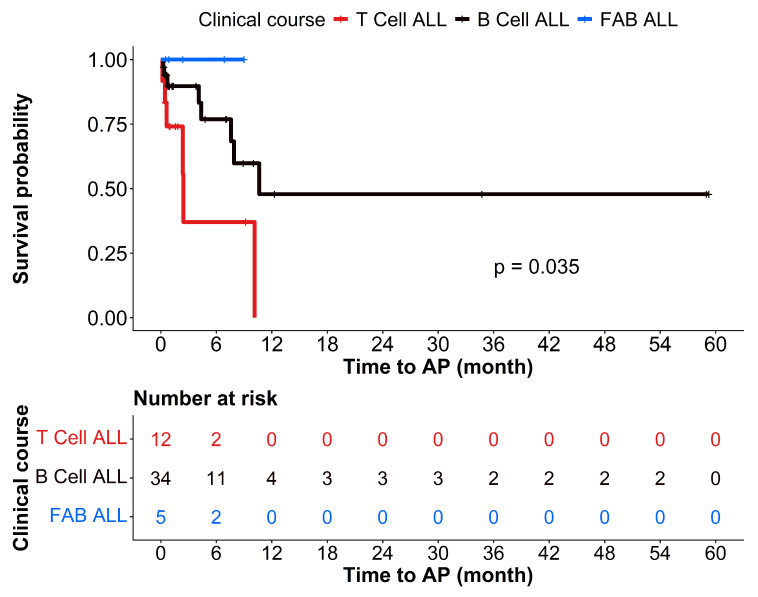
Kaplan–Meier survival analysis indicating that T-cell acute lymphoblastic leukemia (T-cell ALL) had the worst prognosis compared with B-cell ALL and French–American–British-classified (FAB)-ALL, with earlier and more frequent progression to AP after chemotherapy (log-rank *p* = 0.035).

There were no significant differences in the baseline characteristics (age and sex) between patients with acute leukemia with and without pancreatitis (Table 1). Children with T-cell ALL who presented with abdominal pain were more likely to develop AP than those with other types of acute leukemia (Table 1). Kaplan–Meier survival analysis showed that children with T-cell ALL were likely to develop AP earlier than other types, most commonly within the first 6–10 months of treatment (Figure 1). Children receiving high- to very-high-risk chemotherapy protocols had a significantly increased risk of developing AP when they presented with abdominal pain (*p* = 0.026; Table 2). In particular, children with AP tended to receive a higher dose of L-asparaginase than children without AP (64,692.5 vs. 34,483, *p* = 0.094; Table 2).

### 3.2. Clinical and Laboratory Profiles

Regarding the clinical and laboratory profiles, patients with pancreatitis presented with fewer days of abdominal pain before diagnosis (median, 1 day vs. 2 days, *p* = 0.031; Table 3) than those without pancreatitis. Compared with the non-AP group, the AP group showed significantly higher diagnostic and peak serum amylase and lipase levels (*p* < 0.001), but lower albumin levels (3.2 ± 0.9 vs. 3.7 ± 0.7 g/dL, *p* = 0.023). These findings are consistent with the established diagnostic criteria for AP and highlight the severity of the inflammatory process in children with AP. The approaches to requesting radiological investigations differed significantly between patients with acute leukemia with and without pancreatitis (Table 3). Imaging was performed in all patients diagnosed with AP; however, only half of the non-AP patients underwent radiologic investigation (*p* = 0.003; Table 3). CT revealed pancreatic swelling in 78.6% of the patients with AP who underwent the examination. USG showed peripancreatic swelling and collection in 42.9% and 28.6% of the patients with AP who underwent the scan, respectively.

### 3.3. The Management of Acute Pancreatitis

Regarding AP management, almost all patients with AP required nil per os (NPO) at diagnosis (82.4% vs. 24.5%, *p* < 0.001), and the fasting duration was longer than in those without pancreatitis (median 3 days vs. 0 days, *p* < 0.001; Table 3). Children with AP also required significantly higher fluid volumes in the first 48 h (5675.4 mL vs. 3271.6 mL, *p* = 0.003). Opioid and fentanyl requirements were higher in the AP group (Table 3). Among the 17 patients with AP, L-asparaginase was introduced in three of them after AP resolved, all of whom had grade 2 AP. Among these three patients, one subsequently developed persistent AP (walled-off necrosis) following L-asparaginase administration, requiring additional intervention. Importantly, the overall mortality rate (all-cause during follow-up) was significantly higher in the patients with AP than in those without pancreatitis (76.5% vs. 34.0%, *p* = 0.005).

### 3.4. Risk Factors of Acute Pancreatitis

Logistic regression analysis adjusted for potential confounders, including age, sex, and chemotherapy protocol, showed that using a high- to very-high-risk chemotherapy protocol was the only factor contributing to AP development in children with acute leukemia (Table 4). From the ROC analysis, the L-asparaginase accumulative dose of ≥55,200 IU/m^2^ could predict the risk of pancreatitis [Odds ratio = 5.25 (1.29, 24.67), *p* = 0.025] with an area under the ROC curve of 0.695 and a sensitivity of 66.67%, a specificity of 72.4%, a positive predictive value of 50.0%, and a negative predictive value of 84.0%.

Since patients with T-cell ALL may require more intensive treatment, a subgroup analysis was performed in 23 children with T-cell ALL and B-cell ALL who received comparable intensity therapy (high-risk protocol). Among patients presenting with abdominal pain suggesting AP, 16 had B-cell ALL and 7 had T-ALL. AP developed in 6 of 16 patients with B-cell ALL and in 4 of 7 patients with T-cell ALL (*p* = 0.796). In AP cases, the mean cumulative L-asparaginase dose tended to be higher in patients with T-cell ALL than in those with B-cell ALL, although the difference was not statistically significant (83,039 ± 75,709.6 vs. 77,799.8 ± 38,356.4 U/m^2^, *p* = 0.896). Furthermore, the mean interval from leukemia diagnosis to AP onset (3.9 ± 4.3 vs. 4.6 ± 4.1 months, *p* = 0.786) and the timing of L-asparaginase administration were also not significantly different between the two subtypes (21.0 ± 26.2 vs. 28.0 ± 35.1 days, *p* = 0.751).

## 4. Discussion

In this study, the overall prevalence of AP among children diagnosed with acute leukemia was 2.8%, which is consistent with a previously reported prevalence ranging from approximately 2.3% to 18% in pediatric ALL populations, particularly in association with asparaginase-containing chemotherapy regimens [2,7,8,9,10,11].

Importantly, children with T-cell ALL who presented with abdominal pain were more likely to develop AP than those with other leukemia subtypes. This observation aligns with those in prior studies [9,10,31] suggesting that certain treatment-related factors, such as treatment under high- to very high-risk chemotherapy protocols [9,31], as well as leukemia subtype, were associated with AP occurrence [10]. The significantly increased risk of pancreatitis highlights the importance of vigilant monitoring and early diagnostic intervention, particularly in children with T-cell ALL during the first 6–10 months of chemotherapy. We found that using high- to very high-risk chemotherapy protocols was the most significant risk factor for AP in children with acute leukemia.

Knoderer HM et al. reported that the development of pancreatitis associated with L-asparaginase is unpredictable [32] and may be related to genetic susceptibility [33]; nevertheless, we additionally proposed a cumulative L-asparaginase dose cut-off value of ≥55,200 IU/m^2^ to help predict the risk of developing AP in pediatric patients with acute leukemia. This was different from the previously reported cut-off intensity of >45,000 U/m^2^/month as a predictor of AP development [9]. Additionally, Rank CU et al. suggested that L-asparaginase reintroduction should be considered only in patients at a high risk of leukemic relapse [34]. In our study, three patients were readministered L-asparaginase after AP resolution, all of whom were receiving high- to very-high-risk chemotherapy. Only one patient developed persistent AP (walled-off necrosis) following L-asparaginase administration, which may be attributable to specific genetic risk factors [33]. However, genetic testing for predisposition to asparaginase-induced pancreatitis was not performed in this study.

From a clinical perspective, children with AP exhibit shorter durations of abdominal pain before diagnosis than those without pancreatitis. This finding may reflect the more abrupt and severe inflammatory response characteristic of pancreatitis, prompting earlier diagnostic evaluation. Consistent with established diagnostic criteria, patients with pancreatitis demonstrated significantly higher diagnostic and peak serum amylase and lipase levels, as well as hypoalbuminemia, suggesting a more intense systemic inflammatory process. Radiological evaluation plays a crucial role in confirming the diagnosis of pancreatitis. All patients diagnosed with pancreatitis underwent imaging studies, whereas only half of the non-pancreatitis group underwent radiologic investigations [4]. CT revealed pancreatic swelling in most cases of pancreatitis, while USG frequently revealed peripancreatic swelling and fluid collections. Similarly, the importance of imaging in identifying pancreatic edema, necrosis, or complications such as pseudocyst formation, particularly in severe cases, has been previously emphasized [5,35]. The significantly different imaging utilization between groups likely reflects higher clinical suspicion and disease severity in patients with pancreatitis.

Management strategies also differ substantially between patients with and without pancreatitis. Almost all patients with pancreatitis required bowel rest at diagnosis and remained on an NPO for a significantly longer duration. They also received greater volumes of intravenous fluids within the first 48 h, consistent with the guideline-recommended supportive management of AP aimed at maintaining intravascular volume and mitigating systemic inflammation. Additionally, opioid requirements were higher in the pancreatitis group, reflecting more severe abdominal pain and supporting the clinical distinction between pancreatitis-related pain and other causes of abdominal discomfort during leukemia treatment [6].

Importantly, the mortality rate was significantly higher in patients with pancreatitis than in those without. Deaths were not necessarily directly attributable to pancreatitis alone; nonetheless, this finding highlights pancreatitis as a marker of severe treatment-related toxicity and overall vulnerability in children with acute leukemia. AP could also affect continued L-asparaginase use in adequate amounts, which may increase the risk of ALL recurrence and shorten the disease-free interval [36]. Previous studies have reported pancreatitis-related mortality rates ranging from 2% to 9%, particularly in severe or necrotizing cases and in patients requiring treatment modification or asparaginase discontinuation [2,12].

This study was limited by its retrospective nature and small sample size; however, it included the largest cohort of children diagnosed with acute leukemia in Southern Thailand. Overall, our findings suggest that AP, although relatively uncommon, represents a clinically significant complication in children with acute leukemia, particularly among those receiving intensive chemotherapy regimens. Early detection of abdominal pain, prompt laboratory evaluation, and appropriate imaging are essential to ensure timely diagnosis and management. Given the association between pancreatitis and adverse outcomes, heightened vigilance is warranted in high-risk patients, especially those with T-cell ALL or receiving high-intensity treatment protocols with an accumulative L-asparaginase dose ≥55,200 IU/m^2^.

## 5. Conclusions

The risk factors for AP development include using high- to very-high-risk chemotherapy regimens and certain types of acute leukemia. Therefore, early diagnosis and prompt supportive care are essential to reduce complications. Further multicenter studies with larger sample sizes are needed to confirm these risk factors and improve leukemia treatment outcomes in children.

## Figures and Tables

**Table 1:** Clinical characteristics of acute leukemia children presenting with abdominal pain, divided by leukemia subtype (*n* = 70).

Variables	B-ALL (*n* = 34)	T-ALL (*n* = 12)	FAB-ALL (*n* = 5)	AML (*n* = 19)	*p*-Value
Sex—Male, *n* (%)	13 (38.2)	5 (41.7)	2 (40.0)	9 (47.4)	0.947
Age, years (mean, SD)	8.8 ± 4.5	10.7 ± 5.2	8.3 ± 3.6	11.4 ± 3.8	0.152
Race—Thai Buddhism, *n* (%)	21 (61.8)	3 (25.0)	4 (80.0)	16 (84.2)	0.007
BMI, kg/m^2^ (median IQR)	14.8 (13.7, 17.7)	16.2 (13.4, 17.8)	13.5 (13.2, 15.0)	19.3 (16.4, 22.6)	0.001
Phase of chemotherapy					0.130
- Induction, *n* (%)	20 (58.8)	7 (58.3)	2 (40.0)	13 (68.4)	
- Consolidation, *n* (%)	3 (8.8)	4 (33.3)	2 (40.0)	5 (26.3)	
- Maintenance, *n* (%)	8 (23.5)	1 (8.3)	1 (20.0)	0	
- Unknown, *n* (%)	3 (8.8)	0	0	1 (5.3)	
Had prednisolone, *n* (%)	23 (67.6)	11 (91.7)	4 (80.0)	8 (42.1)	0.023
Had vincristine, *n* (%)	28 (82.4)	9 (75.0)	4 (80.0)	4 (21.1)	<0.001
Had doxorubicin, *n* (%)	19 (55.9)	9 (75.0)	3 (60.0)	8 (42.1)	0.355
Had cytarabine, *n* (%)	6 (17.6)	4 (33.3)	2 (40.0)	17 (89.5)	<0.001
Had L-asparaginase, *n* (%)	28 (82.4)	10 (83.3)	3 (60.0)	1 (5.3)	<0.001
Diagnosis of acute pancreatitis, *n* (%)	8 (23.5)	6 (50.0)	0	3 (15.8)	0.114
AP severity by CTCAE, *n* (%)					0.107
- Grade 1	0	0	0	0	
- Grade 2	3/8 (37.5)	3/6 (50.0)	0	0	
- Grade 3	3/8 (37.5)	1/6 (16.7)	0	3/3 (100)	
- Grade 4	0	1/6 (16.7)	0	0	
- Grade 5	2/8 (25.0)	1/6 (16.7)	0	0	
Leukemia outcome: Death, *n* (%)	16 (47.1)	5 (41.7)	2 (40.0)	8 (42.1)	0.979
**Presentation**					
- Abdominal pain, *n* (%)	34 (100)	12 (100)	5 (100)	19 (100)	1.000
- Vomiting, *n* (%)	10 (29.4)	6 (50.0)	2 (40.0)	11 (57.9)	0.179
- Fever, *n* (%)	18 (52.9)	6 (50.0)	2 (40.0)	14 (73.7)	0.359
Duration of abdominal pain before diagnosis, days (median, IQR)	1.0 (1.0, 5.0)	1.0 (1.0, 3.5)	1.0 (1.0, 2.0)	1.0 (1.0, 4.5)	0.867
**Laboratory findings**					
Amylase level at diagnosis, U/L (median, IQR)	75.0 (41.5, 192.0)	133.0 (67.0, 290.0)	52.5 (45.5, 63.2)	39.0 (28.5, 97.0)	0.188
Peak amylase level, U/L (median, IQR)	75.0 (41.5, 192.0)	153.0 (68.5, 272.5)	52.5 (45.5, 63.2)	39.0 (28.5, 97.0)	0.132
Lipase level at diagnosis, U/L (median, IQR)	19.0 (12.0, 175.5)	122.0 (12.0, 468.0)	25.0 (17.0, 65.0)	14.5 (8.0, 138.5)	0.839
Peak lipase level, U/L (median, IQR)	20.5 (12.0, 223.8)	70.0 (35.5, 378.0)	25.0 (17.0, 65.0)	39.0 (8.0, 138.5)	0.613
WBC, ×10^3^/µL (median, IQR)	2.3 (0.8, 4.1)	1.3 (0.9, 3.9)	1.4 (1.1, 2.3)	0.4 (0.1, 2.6)	0.091
Serum LDH, U/L (median, IQR)	433.5 (323.2, 706.0)	546.0 (364.0, 853.2)	369.0 (344.0, 394.0)	330.5 (291.5, 547.8)	1.000
Serum calcium, mg/dL (median, IQR)	9.0 (8.2, 9.5)	9.1 (8.6, 9.3)	8.3 (8.1, 8.6)	8.7 (7.7, 9.2)	1.000
Serum albumin, g/dL (mean, SD)	3.5 ± 0.7	3.6 ± 0.9	4.3 ± 1.0	3.5 ± 0.9	0.360
**Management**					
NPO, *n* (%)	16 (47.1)	6 (50.0)	1 (20.0)	4 (21.1)	0.170
Fluids in first 48 h, mL (mean, SD)	3776.4 ± 2828.1	3839.6 ± 3013.6	1705.8 ± 1524.7	5313.8 ± 1779.4	0.144
Analgesics, *n* (%)					
- Fentanyl	17 (50.0)	4 (33.3)	1 (20.0)	9 (47.4)	0.563
- Morphine	5 (14.7)	3 (25.0)	1 (20.0)	4 (21.1)	0.844
- Paracetamol	19 (55.9)	6 (50.0)	3 (60.0)	9 (47.9)	0.907

AP: Acute pancreatitis, CTCAE: Common terminology criteria for adverse events, WBC: White blood cell count, LDH: Lactate dehydrogenase, NPO: Nil per os, fluids in the first 48 h: total intravenous fluid intake (mL) within the first 48 h following the presentation of abdominal pain.

**Table 2:** Demographic data and treatment protocol in acute leukemia children with and without acute pancreatitis (*n* = 70).

Variables	AP (*n* = 17)	Non-AP (*n* = 53)	*p*-Value
Sex–Male, *n* (%)	7 (40.2)	22 (41.5)	1.00
Age, years (mean, SD)	11.5 ± 4.4	9.3 ± 4.4	0.076
Race—Thai Buddhism, *n* (%)	8 (47.1)	36 (67.9)	0.207
BMI, kg/m^2^ (median IQR)	17.8 (14.5, 19.3)	15.8 (13.9, 19)	0.511
Leukemia subtype			0.116
- ALL T cell, *n* (%)	6 (50.0)	6 (50.0)	
- ALL B cell, *n* (%)	8 (23.5)	26 (76.5)	
- FAB-ALL, *n* (%)	0 (0)	5 (100.0)	
- AML, *n* (%)	3 (15.8)	16 (84.2)	
Chemotherapy protocol			0.026
- High/very high-risk, *n* (%)	13 (76.5)	22 (41.5)	
- Other, *n* (%)	4 (23.5)	31 (58.5)	
Phase of chemotherapy			0.898
- Induction, *n* (%)	9 (52.9)	33 (62.3)	
- Consolidation, *n* (%)	4 (23.5)	10 (18.9)	
- Maintenance, *n* (%)	3 (17.6)	7 (13.2)	
- Unknown, *n* (%)	1 (5.9)	3 (5.7)	
Had prednisolone, *n* (%)	10 (58.8)	36 (67.9)	0.693
Had vincristine, *n* (%)	11 (64.7)	34 (64.2)	1.000
Had doxorubicin, *n* (%)	9 (52.9)	30 (56.6)	1.000
Had cytarabine, *n* (%)	6 (35.3)	23 (43.4)	0.759
Had L-asparaginase, *n* (%)	12 (70.6)	30 (56.6)	0.460
Accumulative dose L-asparaginase per BSA, IU/m^2^ (median IQR)	64,692.5 (44,953.2, 86,600.2)	34,483 (22,727, 59,024)	0.094
Leukemia outcome: Death, *n* (%)	13 (76.5)	18 (34.0)	0.005

AP, acute pancreatitis; Non-AP: Patients without acute pancreatitis; BMI: Body mass index; ALL: Acute lymphoblastic leukemia; FAB: French–American–British (classification); AML: Acute myeloid leukemia; Accumulative dose of L-asparaginase: Calculated as the mean total cumulative dose of L-asparaginase per BSA from the initiation of treatment until the onset of abdominal pain.

**Table 3:** Clinical presentation, investigations, and management of abdominal pain/pancreatitis in acute leukemia children (*n* = 70).

Variables	AP (*n* = 17)	Non-AP (*n* = 53)	*p*-Value
**Presentations**			
- Abdominal pain, *n* (%)	17 (100.0)	53 (100.0)	1.000
- Vomiting, *n* (%)	10 (58.8)	19 (35.8)	0.164
- Fever, *n* (%)	7 (41.2)	33 (62.3)	0.212
Duration of abdominal pain before diagnosis, days (median, IQR)	1 (1, 1)	2 (1, 5)	0.031
Interval from leukemia diagnosis to the onset of abdominal pain, months (median, IQR)	2.4 (0.5, 7.6)	1.2 (0.6, 4.8)	0.837
The timing of L-asparaginase administration, days (median, IQR)	8 (1.8, 47.8)	4 (1, 14)	0.595
**Laboratory findings**			
Amylase level at diagnosis, U/L (median, IQR)	319.5 (219.0, 527.0)	46.0 (35.0, 70.0)	<0.001
Peak amylase level, U/L (median, IQR)	290.0 (216.0, 717.0)	47.0 (35.5, 74.0)	<0.001
Lipase level at diagnosis, U/L (median, IQR)	666.5 (228.0, 997.2)	13.0 (9.5, 25.5)	<0.001
Peak lipase level, U/L (median, IQR)	885.0 (238.0, 1205.0)	15.0 (10.5, 47.5)	<0.001
WBC, ×10^3^/µL (median, IQR)	1.6 (0.7,4.0)	2.0 (0.6, 3.9)	0.799
Serum LDH, U/L (median, IQR)	614.0 (333.0, 1044.5)	336.0 (319.0, 448.5)	0.237
Serum calcium, mg/dL (median, IQR)	8.6 (8.0, 9.3)	9.0 (8.3, 9.4)	0.606
Serum albumin, g/dL (mean, SD)	3.2 ± 0.9	3.7 ± 0.7	0.023
Any imaging performed, *n* (%)	17 (100.0)	30 (56.6)	0.003
- Abdominal X-ray	6 (35.5)	18 (58.1)	0.227
- US abdomen	7 (41.2)	20 (66.7)	0.164
- CT abdomen	14 (82.4)	15 (48.4)	0.046
Imaging findings			
CT pancreatic swelling, *n* (%)	11/14 (78.6)	0	<0.001
US collection, *n* (%)	2/7 (28.6)	0	0.006
US pancreatic swelling, *n* (%)	3/7 (42.9)	0	0.012
AP severity by CTCAE, *n* (%)			<0.001
- Grade 1	0	0	
- Grade 2	6 (35.3)	0	
- Grade 3	7 (41.2)	0	
- Grade 4	1 (5.9)	0	
- Grade 5	3 (17.6)	0	
**Management**			
NPO, *n* (%)	14 (82.4)	13 (24.5)	<0.001
NPO duration, days (median, IQR)	3 (2, 7)	0	<0.001
Fluids in first 48 h, mL (mean, SD)	5675.4 ± 2313.8	3271.6 ± 2586.4	0.003
Analgesics, *n* (%)			
- Fentanyl	12 (70.6)	19 (358.8)	0.026
- Morphine	9 (52.9)	4 (7.5)	<0.001
- Paracetamol	6 (35.3)	31 (58.5)	0.165
Antacid, *n* (%)	3 (23.1)	25 (58.1)	0.058
Feeding type, *n* (%)			0.040
- Oral regular diet	4 (40.0)	9 (90)	
- Oral low-fat diet	4 (40.0)	0	
- Enteral feeding	2 (20.0)	1 (10)	

AP: Acute pancreatitis, WBC: White blood cell count, LDH: Lactate dehydrogenase, CT: Computed tomography, US: Ultrasonography, CTCAE: Common terminology criteria for adverse events, NPO: Nil per os, fluids in the first 48 h: total intravenous fluid intake (mL) within the first 48 h following the presentation of abdominal pain.

**Table 4:** Logistic regression analysis for factors of acute pancreatitis in children with acute leukemia.

Variables	Crude OR (95% CI)	Adj. OR (95% CI)	*p*-Value (Wald’s Test)	*p*-Value (LR-Test)
Age	1.13 (0.99, 1.30)	1.14 (0.99, 1.33)	0.086	0.072
Sex—Female vs. Male	1.01 (0.34, 3.18)	0.93 (0.28, 3.18)	0.910	0.910
Chemotherapy protocol—HR vs. non-HR	4.58 (1.41, 18.00)	4.73 (1.41, 19.21)	0.017	0.011

OR, Odds ratio; CI, Confidence interval; HR: High to very high risk.

## Data Availability

Data supporting the findings of this study are provided in the article and are available from the corresponding author upon reasonable request.

## Abbreviations

The following abbreviations are used in this manuscript:

ALL	Acute lymphoblastic leukemia
AML	Acute myeloid leukemia
AP	Acute pancreatitis
BMI	Body mass index
BSA	Body surface area
CCG	Children’s Cancer Group
CTCAE	Common terminology criteria for adverse events
CI	Confidence intervals
COG	Children’s Oncology Group
CT	Computed tomography
FAB	French–American–British (classification)
IQR	Interquartile range
LDH	Lactate dehydrogenase
NPO	Nil per os
OR	Odds ratio
ROC	Receiver operating characteristic
SD	Standard deviation
US	Ultrasonography
WBC	White blood cell
